# Multivitamin, mineral, and n-3 PUFA supplementation to reduce aggression among long-stay psychiatric inpatients: a randomized clinical trial

**DOI:** 10.1192/j.eurpsy.2022.515

**Published:** 2022-09-01

**Authors:** N. De Bles, N. Rius-Ottenheim, A. Van Hemert, E. Giltay

**Affiliations:** Albinusdreef 2, Department Of Psychiatry, Leiden, Netherlands

**Keywords:** supplements, nutrition, aggression, psychiatric inpatients

## Abstract

**Introduction:**

Aggression and violent incidents are a major concern in psychiatric inpatient care. Nutritional supplementation was found to reduce aggressive incidents and rule violations in forensic populations and in children with behavioral problems.

**Objectives:**

To assess whether multivitamin, mineral, and n-3 PUFA supplementation would reduce the number of aggressive incidents among long-stay psychiatric inpatients.

**Methods:**

The trial was a pragmatic, multicenter, randomized, double-blind, placebo-controlled study. Data were collected from 25 July 2016 through 29 October 2019 at 8 local sites for mental healthcare in the Netherlands and Belgium. Participants were randomized (1:1) to receive either three supplements containing multivitamins, minerals, and n-3 PUFA or placebo for 6 months. The primary outcome was the number of aggressive incidents using the Staff Observation Aggression Scale – Revised (SOAS-R). Secondary outcomes were the patients’ quality of life, affective symptoms, and adverse events.

**Results:**

In total, 176 participants were randomized (supplements, n = 87; placebo, n = 89). Participants were on average 49.3 years old (SD = 14.5), and 64.2% were male. Most patients had a psychotic disorder (60.8%). The primary outcome of SOAS-R incidents was similar in those assigned to supplements (1.03 incidents per month; 95% confidence interval [CI]: 0.74-1.37) and placebo (0.90; 95% CI: 0.65-1.19), with a rate ratio of 1.08 (95% CI: 0.67-1.74; p = .75). Differential effects were not found in sensitivity analyses on the SOAS-R or on secondary outcomes.

**Conclusions:**

Six months of nutritional supplementation did not reduce aggressive incidents among long-stay psychiatric inpatients.

**Disclosure:**

No significant relationships.

